# Exploring perceived risk for COVID-19 and its role in protective behavior and COVID-19 vaccine hesitancy: a qualitative study after the first wave

**DOI:** 10.1186/s12889-022-12900-y

**Published:** 2022-03-15

**Authors:** Naomi J. Patterson, Valerie A. Paz-Soldan, Richard Oberhelman, Lina Moses, Aubrey Madkour, Thomas T. Miles

**Affiliations:** 1 Social, Behavior and Population Sciences Department, Tulane School of Public Health and Tropical Medicine, New Orleans, LA USA; 2 Tropical Medicine Department, Tulane School of Public Health and Tropical Medicine, New Orleans, LA USA; 3 International Health and Sustainable Development, Tulane School of Public Health and Tropical Medicine, New Orleans, LA USA

**Keywords:** COVID-19, Vaccine acceptability, Protective behavior

## Abstract

**Background:**

The novel coronavirus pandemic (COVID-19) has had severe impacts on morbidity and mortality globally.

**Methods:**

This study was set in rural central Kentucky and included participants recruited from public spaces. Fifteen qualitative interviews about personal experiences during the COVID-19 pandemic were conducted by phone from July 3 to July 24, 2020. Interviews were recorded, transcribed, and coded using a grounded theory approach.

**Results:**

Participants who perceived COVID-19 to be a severe risk tended to have personal health concerns and therefore reported taking protective measures for themselves. A slightly smaller proportion of participants reported taking measures to protect others (particularly family). A minority of participants had an ambivalent attitude towards the risk and only took measures if required. COVID-19 vaccine acceptability was low with most participants expressing concerns regarding their need for a vaccine, safety of this vaccine, the value of personal rights, or future vaccine supply.

**Conclusions:**

Most participants perceived some risk of COVID-19 and took steps to prevent infections in themselves and others. Mandates for mask use in certain locations were additionally useful for those who had an ambivalent attitude towards the risk of illness. There was surprisingly little connection between perceiving COVID-19 risk and a desire for the COVID-19 vaccine. In this setting, vaccine acceptability was low, with vaccine concerns outweighing perceived potential benefits. In conclusion, because the risk was often constructed in terms of worries for themselves and others, the framing of health education materials for protective behaviors in these terms may be effective. Furthermore, future COVID-19 vaccine education should address vaccine knowledge and concerns, such as the need for a vaccine and its safety, and emphasize how a vaccination would reduce their chances of severe disease if they were to get sick.

## Background

The SARS-COV-2 (COVID-19) pandemic has restructured every aspect of American life in ways that are likely to be long-lasting [1–4]. Containment of COVID-19 transmission has relied on testing to identify cases, precautions such as increased hand hygiene and cleaning behaviors, quarantine measures, widespread restrictions including mask-wearing (formerly), and vaccination. These strategies have been promoted by public health organizations and governments alike [5]. While protective behaviors are being promoted, exploring the nuance of how individuals perceive the risk posed by COVID-19 is critical. According to the Health Belief Model, protective behaviors are driven by attitudes and perceptions, including the perception of risk associated with enacting or not enacting the behavior in question [6]: more protective measures tend to be taken when individuals perceive greater risk and vice versa [7].

COVID-19 vaccine development began in January 2020 and advanced rapidly [8]. As of April 2020, there were 115 potential vaccine candidates in several development and testing stages [8]. While facing the reality of extensive protective restrictions and limited therapeutics for reducing morbidity and mortality of the COVID-19 pandemic, the use of protective measures and broad vaccine utilization will be necessary to mitigate the spread of the pandemic [5, 9].

Mitigation of COVID-19 risks requires that COVID-19 vaccines not only be available but also widely accepted. Yet, when it comes to vaccine acceptability, it is important to understand components of vaccine hesitancy. Vaccine hesitancy describes a spectrum of beliefs and attitudes towards vaccines resulting in delayed vaccination uptake and outright refusal [10]. Vaccine hesitancy is complex and is often very specific to time, vaccine, and risk groups [11]. This suggests that barriers to vaccine uptake can be different in any given situation [12, 13]. Exploring how vaccine acceptability or hesitation is shaped is critical to informing a national COVID-19 response.

Widespread vaccine hesitancy can limit vaccine uptake and effectiveness. Considering the declining confidence in Measles, Mumps, and Rubella (MMR) vaccines and subsequent outbreaks across the United States and Europe, in 2019 vaccine hesitancy was declared by the World Health Organization (WHO) as a top threat to global health [14–16] that may lead to limited vaccine coverage and ongoing SARS-COV-2 viral transmission. On June 28, 2020, Dr. Anthony Fauci, the director of the National Institute of Allergy and Infectious Diseases, spoke of the current state of vaccine hesitation in the United States, stating that if many citizens refuse a COVID-19 vaccine, attaining widespread individual immunity leading to herd immunity is unlikely [17]. In the time since Dr. Fauci’s statements, the ongoing emergence of variants of concern and new data demonstrating that prior infection and vaccination may not be sufficient to prevent new infections with SARS CoV-2 in all cases makes herd immunity a much further target than previously hoped [18]. Nonetheless, the marked reduction in severity of disease in vaccinated persons and data showing that booster doses are associated with reduced risk of infection, including reducing hospitalizations leading to an overwhelmed health care system underscore the critical importance of COVID-19 vaccination [18–20]. As of May 2021, about 60% of Americans had received at least one dose of a COVID-19 vaccine and as of February 5, 2022 this number has increased to 76% [21, 22]. However, as vaccine rates seemed to peak in mid-April 2021, vaccine uptake seems to have slowed due partly to the manifestation of vaccine hesitation [23].

Meanwhile, the effects of COVID-19 on patients with pre-existing chronic health conditions exacerbate the true risk within populations [24, 25]. For example, populations in rural areas, such as rural Kentucky, tend to have high rates of obesity, diabetes, and hypertension compared to other regions of the U.S. [25, 26]. Patients with hypertension or diabetes are more likely to experience severe cases of COVID-19 [27]. At the same time, those with body mass index measures (BMI) greater than 25 were nearly four times as likely to die and eight times as likely to need advanced respiratory support as a result of their COVID-19 infection [28, 29].

COVID-19 vaccine hesitation is more prevalent in rural areas than in urban areas [30–34], potentially prolonging the impact of the COVID-19 pandemic in those areas [32]. Furthermore, early inconsistency in communication concerning COVID-19 and preexisting vaccine hesitancy may more generally contribute to distrust in COVID-19 vaccines [35, 36]. Considering this, studying COVID-19 vaccine acceptability among rural populations in the United States is of critical importance.

Regarding Kentucky specifically, vaccination rates declined in March 2021, and by February 5, 2022, 6 4% had received one dose, 55% are fully vaccinated, 23% fully vaccinated with a booster/additional dose of a COVID-19 vaccine [31, 32, 37]. This difference between rural and urban areas was about 14% between December 2020 and August 2021 [38]. Vaccine acceptability can be thought of as a combination of the perceived risk and perceived severity of the infection, with the perceived benefit of vaccination [7]. For this reason, to prevent morbidity and mortality associated with COVID-19, we examined how COVID-19 risk perceptions are constructed in order to better address the uptake of protective behaviors and COVID-19 vaccine acceptability among rural residents of Kentucky.

## Methods and analysis

### Recruitment and study design

 Participants were recruited by one of the authors with strong ties to the community. Recruitment took place throughout July 2020 in high-traffic public places in rural central Kentucky, including the parking lots of local retail establishments. Potential participants were asked if they would be interested in participating in a study about COVID-19 and if they were between the ages of 18 and 65. Due to the various infection control measures in place at the time of recruitment, a convenience sample was recruited in person, but interviews were conducted via telephone.

 Using this process, 93 people were approached, and of those, 39 people agreed to participate and provided their contact information. In keeping with COVID-19 social distancing safety measures in place at the time, a study team member then reached out to conduct the interview by phone. Fifteen people ultimately were interviewed and were included in the study. Two study team members (NP and TM) conducted semi-structured phone interviews using an interview guide to ensure that three main themes were addressed: COVID-19 risk perceptions, beliefs and attitudes towards protective or preventative measures, and beliefs and attitudes towards future COVID-19 vaccines. Drawing from the Health Belief Model, questions within the COVID-19 risk perceptions theme sought to explore factors affecting individual perceptions of susceptibility to and severity of COVID-19, while questions within the other themes explored perceptions of benefits and barriers to each behavior, including notions of perceived safety and efficacy [6]. Interviews lasted between 30 and 45 min and provided sufficient data to approach redundancy and saturation. The recorded personal contact information (phone numbers and addresses for compensation) was disposed of after three failed attempts to contact the respondent or the completion of the interview and delivery of their incentive in accordance with the approved protocol.

### Analysis

In-depth interviews were recorded and transcribed verbatim using pseudonyms. Each transcript was coded using inductive thematic content analysis utilizing the analysis software Dedoose [39–41]. Initially, three transcripts were double coded by the two team members who conducted the interviews, creating multiple initial code lists. To develop a working codebook, the coders then discussed and compared these initial lists to identify redundancies, categories, and subcategories of emergent themes. Discrepant codes were discussed and resolved by consensus, and decision trials were noted. This codebook was then applied to each successive transcript by one evaluator, with modifications to the codebook retroactively applied to prior transcripts. Main themes and categories are reported here, with relevant participant excerpts presented in-text.

### Ethical considerations

Informed consent was given before each interview, and participants were compensated for their time. This study was conducted in accordance with the Belmont Principle guidelines for human-subjects research. The study design was approved by the Tulane University Institutional Review Board (IRB) (protocol 2020-738).

## Results

Fifteen interviews were conducted in total. Ten participants were women, and five were men. The average age of the participants was 43, ranging from 27 to 65 years old. All participants were residents of rural Kentucky. Participants were employed by local businesses, retired, and some (*n* = 3) were unemployed at the time of the interview. The themes, categories, and subcategories that emerged from the data are provided here, where “I” indicates the interviewer, “P” the participant, and the information in parenthesis indicates presumed gender, age, occupation.

At the time of data collection, the COVID-19 morbidity and mortality in central rural Kentucky were low. Three of the 15 participants knew of someone personally who had been sick. One participant described it like this:

“I- Why do you think that those people who aren’t following recommendations are making that choice?

P- Um, because it’s not hitting close enough home. And like some of these big cities, the death rate and what they have heard, they might see 3 or 4 or 10 cases in these little small areas. Um, you know, compared to [bigger county] that’s got like hundreds a day.“ (W, 33, Nurse).

The health-related toll contrasted with the microeconomic toll where participants mentioned job losses for themselves and others that they know personally. Where jobs were kept, the workload was altered, sometimes significantly. These contextual specifics may have led to differences in the perceptions of risk and the acceptability of a future vaccine for COVID-19 between localities. Regarding perceived risk, the determination of risk seemed to be dictated by an individual’s close social networks, illustrated by the quotes below.

### Perceived risk associated with COVID-19

All of the study participants (*n* = 15) were aware of COVID-19 and acknowledged that COVID-19 has been causing sickness and death. It was a common understanding that many people had been affected, many have died, and there have been profound economic impacts. One participant described this:

“ I- What does the pandemic mean to you? […]

P- It makes me like feel bad because I don’t like [COVID-19] to happen to my family. Like […] I just feel bad for them.“ (M, 40, Factory worker).

In general, based on the interview context, there was increased concern about COVID-19 over time. From when COVID-19 first emerged, to the time of the interview, perceptions seemed to evolve, though it was often difficult for participants to pinpoint when their perceptions changed. When the threat was perceived as severe, the recommended protective behaviors were also seen as beneficial. The temporality of risk was described as follows by one participant:

“I: you’re talking about when you first heard of it, maybe in February or March and the way you thought of it then. Is that different from how you think of it now? […]

P: So you know between February and April [pause] you know as they if they could quarantine those people that came in from other countries to try to [pause] get a grasp on this pandemic. But they didn’t, because back then, everyone just thought that pandemic was a joke.“ (M, 56, Store worker).

### Levels of concern

In general, there were three main types of risk perceptions found: personal health concerns leading to the utilization of protective behaviors, interpersonal concern leading to protective behaviors, and little or no concern resulting in only implementing the mandated measures.

#### Personal level concerns

The utilization of protective behaviors for personal protection was common. Most participants (*n* = 10) took these measures with their health in mind. In this case, taking the time to do additional handwashing and cleanings, wearing masks, taking social distancing measures, and any other precautions to keep oneself safe was deemed to be the obvious choice. Interviewees who had personal concerns also indicated that taking these measures is now a part of normal life.

For example, this reasoning was described by some as follows:

“I: Does [the way the pandemic is going now] affect how you think about how you are doing things? […]

P: Yeah, you got to think about what’s good for you. I mean, okay, you know it ain’t good for you to walk out in front of a damn bus, don’t you?

I: [laugh] Yes.

P: Yes. Don’t take no rocket scientist to figure out some things.“ (M, 65, Retired).

“I- What do you think about the coronavirus pandemic now and the states re-opening? […]

P- We have to adapt to the virus. Not the virus adapting to us. So, it’s all a matter of finding the way the best way to do what you used to do every day and go with it.“ (W, 29, Education).

#### Interpersonal level concerns

For some (n = 8), the use of protective behavior and materials was motivated by interpersonal concerns. People reported wearing a mask, increased cleaning, and adhering to social distancing measures to keep someone else from getting sick, particularly children and older adults. This was described as follows:

“I: But nowadays, do you wear a mask when you leave the house? […]

P: Yeah. I don’t like them like, I can’t breathe when I have them on because I smoke cigarettes. So it’s like, you really can’t breathe very well when you have them on. But, I mean, in order to keep my daughter from getting sick, I’ll definitely struggle a little bit, you know.“ (W, 37, Unemployed).

#### Minimal to no concern

Other participants did not seem to perceive much of a risk at all (*n* = 5). They may have used protective measures, but only because of local government, workplace, or store requirements. This was often marked with an ambivalent attitude towards the risk of COVID-19 in terms of morbidity and mortality that was generally based on religious beliefs.

“I: Is the way you think about [COVID-19] now, the same as when you first heard about it? […]

P: Yeah. Yeah, I’m not really concerned. I know if I die, I know where I’m going so.

I: What do you mean?

P: Well, if I already get get coronavirus, you know, I’m a Christian. So if I were to get it and not survive it, I would be in heaven. So it doesn’t bother me. You know if I were to get sick.“ (W, 43, Unemployed).

Furthermore, the perception of risk was at times influenced by the individual’s opinion of government or the media, if either positive or negative (*n* = 8). Those who had a negative attitude towards the government or media tended to take minimal precautions. On the other hand, when perceptions about government or media were more positive, more protective measures were taken in general. However, most participants (*n* = 12) did not indicate strong feelings about the government or media to influence decision making.

“I: What do you really think about [the recommendations you hear]?

P: Our politicians are a bunch of morons.

I: Okay, so you mean all of them? Your governor?

P: I’m I’m I’m talking about the 99.9% of the majority of the morons.“ (M, 56, Store Worker).

As opposed to the following sentiment.

“I: Do you think the recommendations and reports are overblown? […]

P: Oh, no no. I wore one [a mask], no I wore one ever since it came ever since it came out that they said that we were supposed to, I’ve I’ve always worn one.

I: When you say they’ve said you’re supposed to, where did you hear that from? The news?

P: Yeah, when the President was having the briefing on TV every day and then Kentucky governor. Yeah, came on and told us that, you know, [COVID-19] was out there, it would be better if people wore the mask. Some people do and some people don’t.“ (W, 58, Retired).

### Vaccine acceptability

While most participants perceived some risk associated with a COVID-19 infection and there were some worries that the pandemic would never end, the use of a COVID-19 vaccine was met with a high level of hesitation. This hesitation centered on four main themes: vaccine knowledge, an individual perceived need for a COVID-19 vaccine, vaccine safety, and infringement on personal liberties. These themes along with other COVID-19 vaccine acceptability findings are discussed here.

#### Vaccine knowledge

Several participants had difficulty understanding the role vaccines would play in preventing COVID-19, frequently confusing vaccines with some form of treatment or cure instead of prevention (*n* = 6). This was expressed in the context of asking questions about vaccines, revealing some misunderstandings that required the interviewers to explain the preventative nature of vaccinations.

“I: What have you heard about the efforts to create a new vaccine for COVID-19? […] Have you heard anything?

P: About the cure?

I: Uh, yeah, about specifically a vaccine.

P: Uh [sigh] Huh? No comment. [laugh]

[…]

I: A cure, is a medicine that you take after you’ve been infected, where as a vaccine you would take it as a healthy….

P: Before you get it?

I: Before you get it.

P: Yeah.

I: In order to prevent it.

P: Alright.“ (W, 27, Factory Worker).

#### Perceived vaccine needs

Almost half of the participants (*n* = 7) did not see the need for a vaccine to prevent COVID-19 for resuming a normal life. They indicated they would not need a reason for vaccine refusal, and the integrity of the information they have received was in question. This was discussed as follows:

“I: All right then on a scale of 1 to 10, with 1 being not at all and 10 being definitely, how likely are you to take a vaccine for coronavirus?

P: Zero.

I: All right. Very clear. Um cool. Can you talk to me a little bit about why this is zero?

P: I just, I just don’t believe in all the vaccines and all this stuff that, I just, I don’t know, I just don’t think that it’s something that I would need to do.“ (W, 43, Unemployed).

“I- Yeah, um, okay. How do you feel about possibly taking a COVID vaccine, COVID-19 vaccine yourself? […]

P- No. I don’t do. I don’t need that. […] Because my wife is here. […] My wife is my medicine.“ (M, 40, Factory Worker).

“I- What would you have to hear in order for you to feel better about getting the vaccine? […]

P- [sigh] Uh. I don’t know if I would trust in it. I guess it has to come, an actual, personal who’s who’s involved with it. […] I mean I’ve been reading articles on Facebook and certain media, and even putting on CNN said that. It’s hard to believe any of them anymore because anything could be tote-up and have a label on it.

I- Yeah.

P- So it would really take, be up to a doctor to truly know [phone ringing in background] to get the right source.“ (W, 33, Nurse).

While the belief that the vaccine was unneeded was common, a minority (*n* = 4) of participants were enthusiastic about the opportunity to be vaccinated.

“I: On a scale of 1 to 10, with 1 being not at all, and 10 being definitely, how likely would you be to take a vaccine for COVID-19 tomorrow if there were one available?

P: A 10.

I: A 10? You would definitely take it?

P: Yes.

I: […] What exactly would motivate you to get it?

P: I just fear for myself. Like especially because I’m out here on my own. […] No family to run to, so I definitely would take that as soon as possible. If I hear about it, I’m going to run for it.“ (W, 27, Factory Worker).

Among these individuals, however, was a concern about the equitable delivery of a vaccine and concerns about possible future vaccine supply (*n* = 4).

“I- Is anything [about a new COVID-19 vaccine] of concern to you?

P- Yes, I am worried. What if it’s only enough for like 20% uh of the community? What if just 80% of the people get it? And what of the homeless?“ (W, 27, Factory Worker).

#### Perceived vaccine safety

It was common that safety worries were perceived as barriers to vaccination and a driving force for vaccine hesitation. At times safety concerns were voiced generally. One participant referenced it like this:

“I-How do you feel about people getting the vaccine?

P- Hey, you can use whatever you want to. It’s your body, you feel like you want to use it, use it. You want to put a bomb in your backseat that’s fine with me. I don’t live with you.

I- Uh ok. So safety?

P- [laughter] You asked, I answered.“ (M, 65, Retired).

In addition to general safety concerns, because the vaccine would be newly developed, the worries about side effects (*n* = 9) and vaccine components (*n* = 7) seemed to be more pronounced than they would be otherwise. Many participants (*n* = 9) pointed to unknown side effects as a major concern.

“I- So on a scale, of let’s say 1 to 10 again, with 1 being kind of not at all, 10 being definitely, how likely would you be to take a to take the vaccine for COVID-19 tomorrow if it was approved and said to be safe, and it was available?

P- uh. I probably wouldn’t.

[…]

I- Why, why do you think that is? […]

P- Just, there’s no knowing the long-term effects. They won’t know those until they do a mass, you know, mass doses to everybody. It might be 2 or 3 years later they start realizing things that that come out of it.“ (W, 33, Nurse).

Similarly, one participant described vaccine content worries as follows.

“I: Um. And, ah what about safety? Are you concerned at all about the safety of a vaccine that they might make for covid?

P: I am. I actually am. But what if they [pause]. Uh, how do I say it. [pause] What if they’re putting less of what’s in and putting more of what doesn’t need to be in? […]

And what if they’re putting, not the right amount? What if the amounts are uneven of what really needs to be in the vaccine, and they’re putting less of it? You know the wrong amounts.

I: Yeah, yeah. What, what makes you worried about the way they make it?

P: Well are they sanitized? Um. Are they clean? Are like have they washed their hands? Have they make sure that it’s not, you know something that’s open, and they decide to reuse it.“ (W, 27, Factory Worker).

#### Perceived Infringement on Personal Liberties

Vaccine concerns in general and concerns that potential vaccine mandates may infringe upon personal rights made a vaccine for COVID-19 undesirable. Mandates were occasionally spoken about positively. For example, the quote below suggests that only a mandate could prompt the participant to be vaccinated.

“I: Yeah. So, if the vaccine was available, say, tomorrow. Would you take it?

P: Uhh. That would be an easy question. Probably. If it was, if it was mandatory that you had to take it. I probably would. Um, I don’t don’t work. So, I’m retired and we’re not out of the house a whole lot. So, it’s kind of like when a doctor tells you that you need a flu shot, and then then she says, Well, if you’re really not in the public a lot, I don’t really advise you to take one. So that would probably be where I would stand. And I’d, I’d, I don’t, I probably wouldn’t take it unless it was mandatory that I had to take it for uh some reason.“ (W, 58, Retired).

However, about half of the participants (*n* = 7) were against vaccine mandates for COVID-19, as well as other vaccines more generally, implying that a vaccine mandate would take away liberties in a way that shouldn’t be allowed in the United States. Beyond personal rights potentially being infringed upon, it was also sometimes implied that desiring a mandated vaccine would be foolish.

“I: Then how do you feel about mandatory vaccines? Having a rule that everybody should get vaccinated?

P: Bullshit. What rule says to fit something into your body? They should not be allowed to force you to put something into your body.

I: About for schools? About for everybody?

P: Everybody. Anybody. We’re supposed to, should have rights and freedoms. [pause] They should not be allowed to force anything. [background noise]

I: Um, can I ask you why?

P: Because it’s taking away our rights. And that’s what makes this America.“ (W, 37, Unemployed).

## Discussion

The perception of risk and associated protective behavior is likely closely tied with rurality, where risk perception and protective behaviors likely differ between rural and urban communities [42]. The connection between the perceived risk of COVID-19 and COVID-19 vaccine acceptability is not overly apparent here, contrary to some similar studies [43–49]. When it comes to the perceptions of risk in the context of the COVID-19 pandemic, the participants universally understood that they might get sick. When considering the temporality of perceived risk, there was inconsistent seriousness paid to the necessity of protection. However, after months of news stories, government regulations, and widespread economic impacts, the community members in this study were aware of protective measures that are recommended to be taken. This change in perception of the seriousness of the pandemic was often intangible when asked if thoughts have changed over time, yet at times marked when the local government or common storefronts ordered mandates for mask use.

The evaluation of the emerging data revealed three main groups of participants in terms of perceived risk: those with personal health concerns, those with interpersonal health concerns, and those with little to no concern. Protective behaviors were most commonly used for personal protection. According to Dryhurst et al., often, these measures were taken for oneself as well as when an individual’s actions could affect a loved one [50]. Focusing on the protection of others is an important aspect of health promotion efforts and the way health warnings were communicated both locally and globally [51, 52]. The emphasis that this behavior is not only for your health but also for protecting others seems to be effective when considering the uptake of protective behavior.

When the participants perceived the risk of contracting COVID-19 to be small or inconsequential, the participants tended to view getting sick as just a part of life. Those individuals tended to have little desire to change how they do things to prevent negative outcomes and only took precautions because they were mandated. It has similarly been shown that when the risk of illness seemed to be small, protective behaviors tended not to be taken [53]. In this participant group – perceiving little or no concern for COVID-19 -- health messaging in terms of personal or interpersonal impacts seemed to be unmoving. This may give credence to the idea that mandates can induce behavior change during a pandemic when other forms of communication did not [54].

When it comes to the COVID-19 vaccine and related mandates, the themes that emerged included a lack of perceived need, safety concerns, and concerns over personal liberties. As a reason for vaccine hesitation, the perceived need seemed to at least partially point to a lack of understanding of a vaccine’s purpose. Understanding the intended benefits of vaccination tended to be associated with increased vaccine acceptance [55, 56]. However, acknowledging a lack of need seemed to be a strong motivator that may not be influenced by vaccine promotion efforts, as these participants seemed unwilling to vaccinate in any circumstance.

In addition to a low perceived need, many who might consider vaccination indicated they would prefer to wait and see if it is safe first. They indicated that they would likely wait until well after the vaccine was licensed and available to take it unless it was required to do so by an employer. This safety concern encompassed potentially dangerous vaccine components and unknown side effects. Many of these concerns seemed rooted in the fact that a COVID-19 vaccine would be new, though some mentioned safety concerns regarding other vaccines as well, regardless of when they were developed. Concerns based on safety, composition, novelty, possible side effects, and the desire to delay vaccination are common tropes in vaccine hesitancy literature. In fact, many of these same concerns were discussed in a 2021 review of factors contributing to vaccine hesitancy during recent pandemics, epidemics, and global outbreaks including the 2009 H1N1 pandemic [49, 57].

Machingaidze et al., in particular suggest that worries surrounding vaccine hesitation globally are complex with several contributing factors [49]. Concern over vaccine safety, vaccine need, and logistical distribution worries indicated here align closely with concerns relating to vaccine hesitancy in low- and middle-income countries [49]. Sociodemographic factors such as political affiliation and location of residence also contribute to high levels of vaccine hesitation in some areas [36].

Nonetheless, mandated vaccination for themselves or their children was often described as unwanted. This is a common occurrence and not specific to a COVID-19 vaccine [13, 58]. Considering how often concerns of future safety were voiced, the possibility that a mandate may force the use of a vaccine that the community had concerns about could lead to general mistrust [59].

The danger of COVID-19 was often evident, and most participants in varying degrees perceived the risk it posed to this population. This, however, did not seem to reliably translate to the desire for a new COVID-19 vaccine. In this instance, vaccine concerns appeared to outweigh the potential benefits of preventing sickness for most participants. There was general unease towards a potential vaccine, and only a minority would be enthusiastic in receiving one regardless of potential concern. This is consistent with the current state of vaccine hesitation, where the outlook is variable, and hesitation may or may not prevent eventual vaccination administration [11, 12, 60]. While desire for a vaccine usually comes along with recognizing the risk associated with future illness, for most participants in this study, the risk of illness as a result of COVID-19 infection did not outweigh the risk they associated with a future COVID-19 vaccine [13].

While perceiving greater risk or seriousness of a COVID-19 infection seemed to be closely tied to enacting protective measures that are less invasive than vaccines among this population, the relationship between perceived risk and acceptability of a future COVID-19 vaccine did not seem to be as strong, suggesting that messaging around the risk associated with a COVID-19 infection may resonate more strongly within this community when targeting preventative behaviors other than vaccination. Low levels of vaccine literacy, concerns around perceived need, safety, and personal liberties underpin why a COVID-19 vaccine may encounter hesitant beliefs and suggest that even in the context of a COVID-19 pandemic, public health officials seeking to promote COVID-19 vaccination should design messaging targeting healthcare provider recommendations, vaccine literacy and more generalized mistrust to address the low perceived susceptibility, safety, and concerns about personal freedoms.

This study adds to a large body of literature exploring COVID-19 risk perception and provides much needed detail about the relationship between those perceptions and protective behaviors, including vaccination in rural areas of the United States [35, 61, 62]. While the results contribute to a growing body of COVID-19 literature outlining factors that influence vaccine hesitancy, results suggesting greater acceptance of other preventative measures among this rural population highlight the differential way risk perceptions shape intention for different protective behaviors, an area of study that is likely to become more important as we think about future pandemics, pandemic preparedness, and the challenge of health communication during infectious disease outbreaks [63]. Considering the elevated awareness of infection control during the pandemic, this study may be poised to contribute to effective health communication specific to vaccination and other protective behavior [56].

This study has several strengths and limitations. First, as with all studies that rely on qualitative data, social desirability bias may have shaped the way participants responded during the interviews. However, the fact that interviews were conducted by phone instead of in-person, face-face, thereby allowing participants to maintain some distance and anonymity from the interviews, likely made it easier for respondents to respond. Secondly, the generalizability of this study is particularly limited. Not only was the sample chosen based upon convenience, which could have led to the introduction of selection bias, but while the study was open to individuals 18-65 years old, our sample skewed older. It didn’t include populations 70 and older who may be more vulnerable to severe cases of COVID-19. Additionally, participant refusal rates were high resulting in a small sample size. Thus, efforts to extrapolate these findings to other rural communities and age groups should be made cautiously despite approaching saturation within this data. Furthermore, as the pandemic continues to evolve, viewpoints on protective behaviors and vaccine acceptability may have evolved since data was collected. Nonetheless, this approach did provide us with rich details about COVID-19 risk perceptions and their relationship to several preventative behaviors or vaccination intentions, using participants’ own words, on a topic that would have been harder to assess in a quantitative study.

## Conclusions

This study has found that in one rural area of the US, perceived risk is expressed in terms of worries for themselves, others, or an ambivalent attitude towards possible outcomes. Increased recommendations by trusted sources or framing interventions in terms of protecting themselves or others may be useful for promoting non-invasive protective behaviors such as wearing masks, social distancing, and increased cleaning. These perceived risks do not seem to correspond with a willingness for a COVID-19 vaccine. As vaccination is necessary to slow spread and reduce hospitalizations that overwhelm the health care system, promoting vaccine acceptability is of critical concern. This vaccine promotion effort may be positively impacted if vaccine safety was addressed, trusted public sources drove the messages, and messages emphasized how vaccinations help prevent the spread of COVID-19 to others and prevent severe disease.

Furthermore, as the pandemic continues to spread and new variants cause additional concern, well-known or personal experiences of sickness may influence individuals to become more accepting of protective behaviors or preventative vaccines. Exploring risk perception and vaccine acceptability using qualitative methods may be useful in guiding communication or policy during future pandemic events. However, as the interviews were conducted before a COVID-19 vaccine was available, the gap between behavioral intention and behavior suggests a need for longitudinal studies which take into consideration additional factors that may influence uptake in other rural [64].

## Data Availability

The data generated and analyzed during the current study are available from the corresponding author on reasonable request.
